# Evaluation and Selection of Candidate Reference Genes for Normalization of Quantitative RT-PCR in *Withania somnifera* (L.) Dunal

**DOI:** 10.1371/journal.pone.0118860

**Published:** 2015-03-13

**Authors:** Varinder Singh, Sunil C. Kaul, Renu Wadhwa, Pratap Kumar Pati

**Affiliations:** 1 Department of Biotechnology, Guru Nanak Dev University, Amritsar-143005, Punjab, India; 2 Cell Proliferation Research Group and DBT-AIST International Laboratory for Advanced Biomedicine, National Institute of Advanced Industrial Science and Technology, AIST, Tsukuba, Ibaraki, 305 8562, Japan; University of Vigo, SPAIN

## Abstract

Quantitative real-time PCR (qRT-PCR) is now globally used for accurate analysis of transcripts levels in plants. For reliable quantification of transcripts, identification of the best reference genes is a prerequisite in qRT-PCR analysis. Recently, *Withania somnifera* has attracted lot of attention due to its immense therapeutic potential. At present, biotechnological intervention for the improvement of this plant is being seriously pursued. In this background, it is important to have comprehensive studies on finding suitable reference genes for this high valued medicinal plant. In the present study, 11 candidate genes were evaluated for their expression stability under biotic (fungal disease), abiotic (wounding, salt, drought, heat and cold) stresses, in different plant tissues and in response to various plant growth regulators (methyl jasmonate, salicylic acid, abscisic acid). The data as analyzed by various software packages (geNorm, NormFinder, Bestkeeper and ΔCt method) suggested that cyclophilin (*CYP*) is a most stable gene under wounding, heat, methyl jasmonate, different tissues and all stress conditions. *T-SAND* was found to be a best reference gene for salt and salicylic acid (SA) treated samples, while 26S ribosomal RNA (*26S*), ubiquitin (*UBQ*) and beta-tubulin (*TUB*) were the most stably expressed genes under drought, biotic and cold treatment respectively. For abscisic acid (ABA) treated samples *18S-rRNA* was found to stably expressed gene. Finally, the relative expression level of the three genes involved in the withanolide biosynthetic pathway was detected to validate the selection of reliable reference genes. The present work will significantly contribute to gene analysis studies in *W*. *somnifera* and facilitate in improving the quality of gene expression data in this plant as well as and other related plant species.

## Introduction

*Withania somnifera* is an important medicinal plant of the family Solanaceae and is extensively used in Indian, Unani and African systems of traditional medicine [1]. It has strong immunomodulatory, anti-stress, cardioprotective, anti-aging, antioxidant, anti-inflammatory, anti-tumour activities and chemo preventive properties [2–6] that have been attributed to its wide secondary metabolites including withanolides, glycowithanolides, alkaloids, flavanol glycosides, sterols and phenols [7–10]. Withaferin A and withanone, in particular, have attracted a lot attention due to their anti-cancer potentials [11–15] and hence identification, cloning and characterization of genes involved in withanolide biosynthesis in *W*. *somnifera* has become a focus of study in several laboratories [16–19]. Studies on dynamics of gene expression involved in withanolide biosynthesis with respect to development, growth conditions, and stress hold great potential as it provides necessary inputs on the levels of target metabolite synthesis and accumulation for medicinal use.

Various techniques like Northern blotting, RNase protection assay, and semiquantitative reverse-transcription PCR are used for gene expression analysis. Quantitative real-time reverse transcription polymerase chain reaction (qRT-PCR) is preferred due to its higher sensitivity, specificity and broad quantification range. However, in spite of these precisions and robusticity, it is often limited by the lack of reliable reference genes for normalization and authenticity of the experimental outcomes. Therefore, identification of stable reference genes for accurate normalization of qRT-PCR results is an important aspect of these studies [20]. In most of the cases, housekeeping genes that are constitutively expressed are selected as reference genes. However, several studies have reported that the expression stability of commonly used housekeeping genes is inappropriate for normalization and exhibit expression variability under different experimental conditions [21–22]. Thus, it is necessary to first validate the expression stability of control genes that are expressed constitutively across experimental treatments prior to their use for normalization of data.

In *W*. *somnifera*, several studies have reported qRT-PCR analysis of gene expression [16, 23, 24]. However, to the best of our knowledge, a systematic study on endogenous control genes expression under different stress conditions has not been accomplished yet. In the present study, a comprehensive gene expression analysis involving 11 genes including 18S ribosomal RNA (*18S-rRNA)*, 26S ribosomal RNA, *(26S)*, Actin *(ACT)*, cyclophylin *(CYP)*, elongation factor-1 *(EF-1)*, glyceral-dehyde-3-phosphate dehydrogenase (*GAPDH)*, ribosomal protein L2 *(RPL2)*, Sand family protein *(SAND)*, alpha-tubulin (*TUA)*, beta-tubulin (*TUB*) and ubiquitin (*UBQ*) was evaluated in different plant organs (flower, leaf, stem, root and whole plant), biotic (fungal disease), abiotic (salt, cold, heat, drought and wounding) and plant growth regulators (methyl jasmonate (MJ), salicylic acid (SA) and abscisic acid (ABA) stress treatments. Three withanolides biosynthetic pathway genes [3-Hydroxy 3-methylglutaryl coenzyme A reductase (*HMGR*), Cycloartenol synthase (*CAS*) and Cytochrome 450 Reductase (*P450*)] were used to validate these results. Top two reference genes singly and in combination were used to test the reliability of selected reference genes. Several algorithms like geNorm, NormFinder and Bestkeeper were used to validate the expression stability of different genes under various experimental conditions.

## Material and Methods

### Plant materials

*W*. *somnifera* seeds were germinated in greenhouse (22–25°C 14h light/10h dark) in earthen pots containing a mixture of soil: sand: vermicompost (1: 1: 8). After 30 days, the germinated plants were transferred to individual pots. Leaves of four months old plant were used for experimentation. To study the tissue specific expression of the 11 candidate genes, different plant organs (Flower, leaves, stem, roots and whole plant) were collected and frozen in liquid nitrogen and stored at -80°C for further experimentation.

### Abiotic stress treatments

For heat and cold treatment plants were incubated at 42°C±1 and 4°C±1 respectively under dim light. Control plants were kept at 25°C. For wounding treatment, leaf surface was scraped with sterile forceps. For salt treatment, plants pots were saturated with 200 mM NaCl solution followed by leaf sampling. For drought treatment of the plants, water was with hold for 7 days after which samples were collected. For non-stress treatment all the plants were watered at regular intervals. Samples were collected as fully expanded leaves after the heat, cold, salt, wounding and drought stress treatments at 0, 6, 12, 24 and 48 h interval of time. All samples were immediately frozen in liquid nitrogen after harvest and stored at -80°C prior to RNA isolation. Two sets of plants were used for different stress treatment (biological replicates).

### Hormone treatments

For hormone treatments, four months old plants were sprayed with methyl jasmonate, salicylic acid, abscisic acid (100 μM each). Control plants were sprayed with distilled water. Samples were collected at 0, 6, 12, 24 and 48 h intervals, immediately frozen in liquid nitrogen and stored at -80°C for further analysis.

### Biotic stress treatment

In *W*. *somnifera*, leaf spot caused by *Alternaria alternata* is one of the most prevalent disease [25]. The isolated pathogen from leaf spot infected plant was used to induce biotic stress. *A*. *alternata* spore suspension of 6×10^5^ spores/ml with 0.01% Twin20 (v/v) was prepared from 7–10 days old fungal cultures in sterile distilled water, and concentration of spores was adjusted by using a haemocytometer. This spore suspension was sprayed on leaves of healthy plant and pots were kept in moist chamber (95±5% Relative Humidity) and maintained at 25°C for 24h required for optimal infection (72 hours). Plants treated with sterile distilled water with 0.01% Twin20 (v/v) in the same manner were used as control. Fully expanded leaves showing disease symptoms (brown spots) in 1/3^rd^ or 1/5^th^ of the leaf area were harvested (immediately frozen in liquid nitrogen and stored at -80°C).

### RNA Extraction and cDNA Synthesis

The sampled plant leaves were grounded to fine powder with mortar and pestle in liquid nitrogen, and 100 mg of the material was used for RNA isolation. Total RNA was extracted using Trizol reagent (Invitrogen, http://www.invitrogen.com), as per the manufacturer’s guidelines. Isolated RNA was treated with RNase free DNase (Sigma-Aldrich, USA) to remove genomic DNA contamination. Purity and concentration of RNA samples was measured using NanoDrop (Thermo Scientific) and integrity was checked on agarose gel electrophoresis. RNA samples with 260/280 ratio between 1.9 and 2.1 were used for subsequent experimentation. First strand cDNA synthesized using iScript cDNA synthesis kit (BioRad) according to manufacturer’s instructions in a total volume of 20 μl containing 2 μg total RNA. RNA extraction and cDNA synthesis from all samples were performed for two biological replicates. The cDNA solution was 10 times diluted with nuclease-free water and aliquots were stored at -20°C till qRT-PCR.

### Primer design and quantitative real-time PCR assays

Gene-specific primers were designed using Primer Quest software of Integrated DNA Technologies (http://www.idtdna.com/Primerquest/Home/Index).using default criterion of the software with amplified products ranging from 75 to 150 bp and Tm around 60°C. Primer sequences used in the qRT-PCR analyses are presented in Table 1. The primers were further validated for unique amplicon using Primer-BLAST (http://www.ncbi.nlm.nih.gov/tools/primer-blast/). Standard RT-PCR was performed for all the primer pairs and a single amplification product of the expected size for each gene was obtained by electrophoresis on a 2% agarose gel. Primer amplification efficiency was determined from standard curve generated by serial dilution of cDNA (10 fold each) for each gene in triplicate. Correlation coefficients (R^2^ values) and amplification efficiencies (E) for each primer pairs were calculated from slope of regression line by plotting mean Cq values against the log cDNA dilution factor in Microsoft Excel using equation E = (10^(1/-slope^−1) X 100. Real-time amplification reactions were performed in 96 well plates using SYBR Green detection chemistry and run in triplicate on 96-wells plates with the StepOne Plus Real-time PCR machine (Applied Biosystems). Reactions were prepared in a total volume of 20 μl containing: 1 μl of 10 fold diluted template, 0.5 μl of each amplification primer (1μM), 10μl of 2X Fast SYBR Green (Applied Biosystems) and final volume of 20 μL with sterile nuclease free water. Non-template controls (NTC) were also included for each primer pair. The cycling conditions were set as default: initial denaturation step of 95°C for 20 sec to activate the Taq DNA polymerase, followed by 40 cycles of denaturation at 95°C for 3s, annealing at 60°C for 30s. The melting curve was generated by heating the amplicon from 60 to 90°C. Baseline and threshold cycles (Ct) were automatically determined using the StepOne Plus Software version 2.3 (Applied Biosystems). All the experiments were done according to MIQE (Minimum Information for Publication of Quantitative Real-Time PCR Experiments) guidelines [26].

**Table 1:** Details of Reference genes and their primer sequences for each of the 11 evaluated genes.

Gene Symble	Primer sequences (5' to 3') FP/RP	Gene description	Accession Number	Amplicon size (bp)	Tm (°C)	E (%)	Regression coefficiant (R^2^)
***18S-rRNA***	TGAGAAACGGCTACCACATC	18S ribosomal protein	AJ236016.1	106	62	98	0.998
	AGACTCATAGAGCCCGGTATT						
***26S***	GAACGGGCTTGGCAGAATTA	26S ribosomal RNA	JQ408705.1	103	62	97	0.996
	CCAGCTCCCACCTATACTACAT						
***ACT***	CTTCCCGATGGTCAAGTCATTA	Actin	X55749.1	116	62	96	0.997
	TTGTATGTGGTCTCGTGGATTC						
***CYP***	AGGTGTTGGAAAGATGGGTAAG	Cyclophilin	AF126551.1	87	62	94	0.991
	TCACCTCCTTGACACATGAAC						
***EF-1***	GGAACTTGAGAAGGAGCCTAAG	Elongation factor 1	X14449.1	107	62	102	1.00
	ATGGAGGGTATTCAGCAAAGG						
***GAPDH***	GTGCTGACTTCGTTGTTGAATC	Glyceraldehyde-3-phosphate dehydrogenase	U97257.1	99	62	102	0.993
	GAGCAGAGATCACAACCTTCTT						
***RPL2***	CCGTCATCCTTTCAGGTACAA	Ribosomal protein L2	X64562.1	119	62	89	0.987
	GCAACACGTTACCAACCATAAG						
***T-SAND***	GGTCAATCCGAGTACGAATCC	Sand family protein	SGN-U316474	90	62	90	0.995
	CGGGAGCTCCTTCAGTTTATC						
***TUA***	CTCTGTGGACTATGGCAAGAAA	Alpha-Tubulin	AJ421411.1	101	62	90	0.996
	TTGACAGGACACTGTTGTAAGG						
***TUB***	TGGTACACAGGTGAAGGAATG	Beta-tubulin	DQ205342.1	102	62	86	0.999
	TGCTGTAGCATCCTGGTATTG						
***UBQ***	AGCTGCTATACTGACTTCAATCC	Ubiquitin	AB026056.1	111	62	94	0.996

Notes: Tm = Annealing temperature, E = PCR efficiency.

### Statistical analysis of gene expression stability

To analyze the expression variation of the ten candidate reference genes, three publicly available software tools, i.e., geNorm version 3.5 [27], NormFinder version 0.953 [28], BestKeeper [29] and Comparative ΔCt method [30] were used. Relative expression of mean Cq values of 6 replicates (2 biological and 3 technical) was calculated by 2^-ΔΔcq^ formula [31]. To find relative expression ΔCt is calculated by subtracting lowest Cq value from other Cq values for each sample and imported into geNorm and NormFinder softwares for stability analysis. For BestKeeper and ΔCt method mean Cq values were used as input.

### Validation of reference genes

For the validation of the selected reference genes for normalization of expression, we examined the effect of salicylic acid on the expression level of three key genes of withanolide biosynthetic pathway such as 3-Hydroxy 3-methylglutaryl coenzyme A reductase (*HMGR*), Cycloartenol synthase (*CAS*) and Cytochrome 450 Reductase (*P450*). The information regarding genes and primers are listed in S1 Table. We selected *T-SAND* and *UBQ* alone and in combination as the reference genes and as combination their geometric mean was used for the expression studies. Further, to analyze the influence on gene expression by selection of inappropriate, we also selected least recommended *GAPDH* gene. *T-SAND* and *UBQ* were found to be most stable genes while *GAPDH* was least stable according to comprehensive ranking for SA treated samples. The expression levels were measured using 2^-ΔΔcq^ method.

## Results

### RNA integrity and expression profiling of candidate reference genes

**Fig 1:**
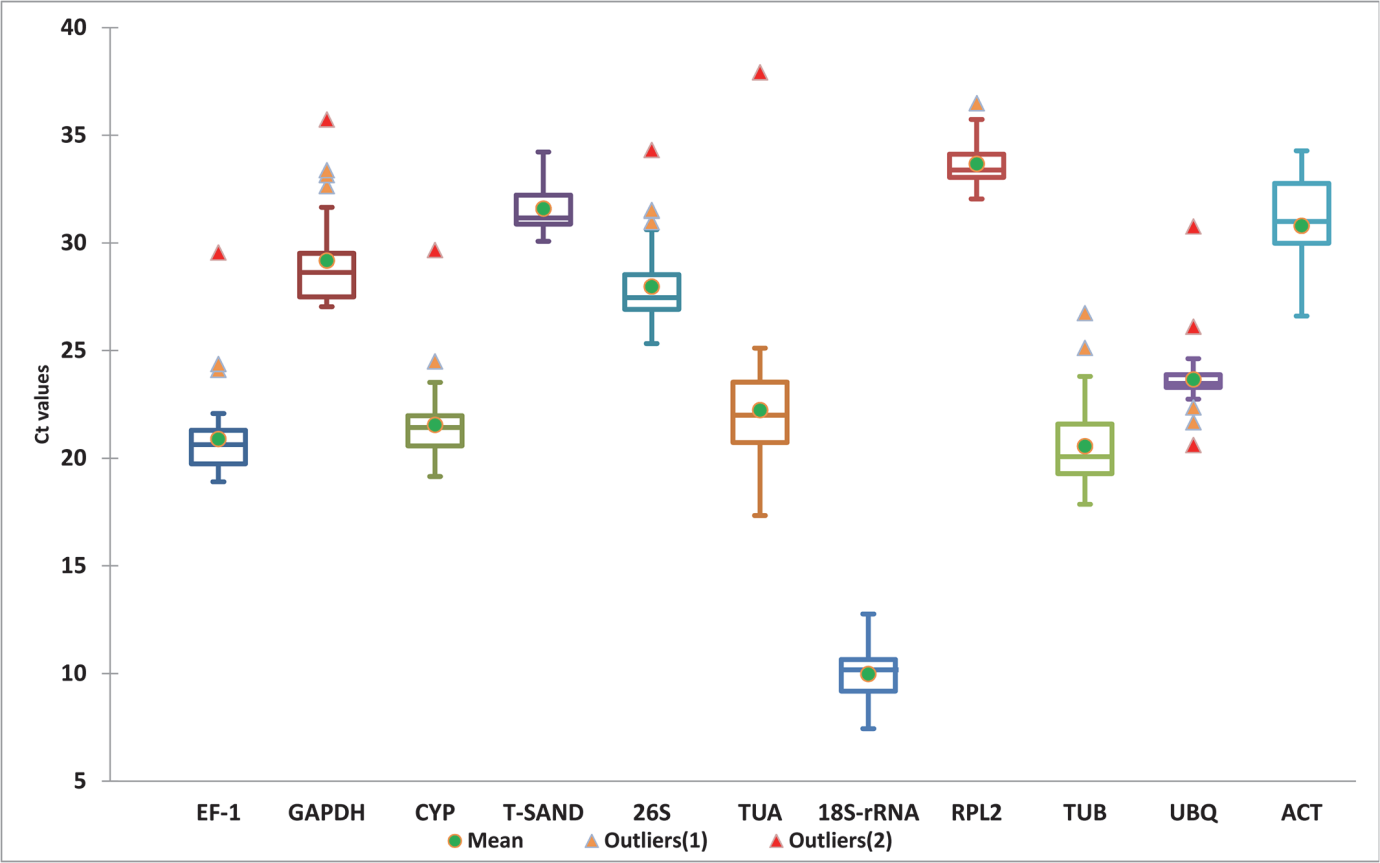
Distribution overview of expression levels (Ct) of the different genes. Boxplot representation of raw Cq values obtained from amplification curves. The box indicates the 25th and 75th percentiles. Whiskers represent the maximum and minimum values, the thin line within the box marks the median, mean (thick dot) and outliers (Δ).

**Fig 2:**
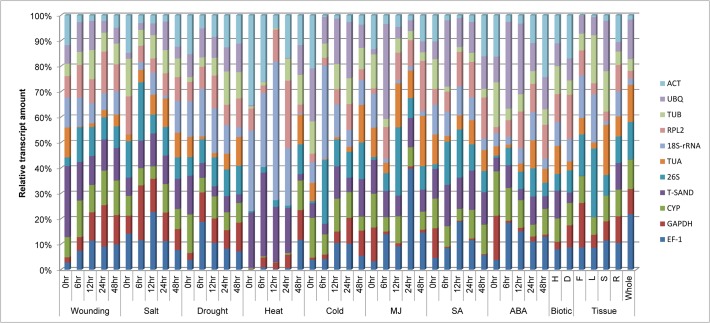
Distribution of transcript populations of 11 selected genes under different stress treatments. In each treatment, the transcript amount of each gene is shown as a ratio relative to the sum of the 11 transcript populations. The samples were collected at 0hr, 6hr, 12hr, 24hr and 48hr interval of time after each treatment. Different treatments were heat, cold, drought, wounding, salt, MJ, SA, ABA, biotic and tissue (Flower, leaf, stem root and whole plant).

Total RNA extracted from leaf samples was evaluated for quality and integrity. RNA integrity was checked on agarose gel electrophoresis (S1 Fig.). Real-time qRT-PCR was conducted with 11 candidate reference genes; 18S ribosomal RNA *(18S-rRNA)*, 26S ribosomal RNA *(26S)*, Actin *(ACT)*, cyclophylin *(CYP)*, elongation factor-1 *(EF-1)*, glyceral-dehyde-3-phosphate dehydrogenase *(GAPDH)*, ribosomal protein L2 *(RPL2)*, Sand family protein *(SAND)*, alpha-tubulin *(TUA)*, beta-tubulin *(TUB)* and ubiquitin *(UBQ)*. The specificity of the designed primer pairs was assessed by a confirmatory PCR and 2% agarose gel electrophoresis that revealed expected single bands of desired lengths (S2 Fig.). Further, the PCR amplify fragments were sequenced to validate specificity of gene primers. Furthermore, specificity was also confirmed from single peak in dissociation curve and without peak in no template control (NTC) which revealed the absence of any genomic DNA (S3 Fig.). PCR efficiency (E) and correlation coefficient R^2^ was estimated from standard curve showed range between 86–102% and 0.991–1.00, respectively (Table 1). The Cq values of the raw expression data across all samples are shown in Fig. 1, which varied from 7.44 to 36.48 and mostly lying between 19–23 range. Among all tested reference genes, *18S-rRNA* showed the highest abundance (lowest Cq value) with a mean of 10.28 followed by *EF-1* (21.11) and *TUB* (21.20), *CYP* (22.06), *TUA* (22.66), *UBQ* (23.59), *26S* (28.48), *GAPDH* (29.19). *RPL2*, *T-SAND* and *ACT* showed the least transcript abundance with a mean Cq of 33.65, 31.83 and 31.79, respectively (Fig. 1). *UBQ*, *RPL2*, *18S-rRNA* and *CYP* showed the lowest gene expression variation (below 4 cycles) while *TUA*, *TUB* and *26S* had highest expression variation (above 7 cycles). Transcript quantities are shown for each treatment as ratios relative to the sum of the all transcript populations. Variations in the relative quantities of the transcripts with different treatments are shown in Fig. 2. Such type variation in the expression level reflects that there is lack of consistency in their expression pattern under experimental stress responses. For example *18S-rRNA* mRNA represents approximately 10% of the total mRNA population in wounding treated samples but more than 40% in heat treatment. We found that the amount of mRNA transcript of all genes varied in all treatment. The transcript level of *CYP* remained relatively constant in wounding, different plant organs and biotic treatment. Similarly transcript level of *T-SAND* remained constant for salt and salicylic acid treatment and those of *RPL2* were also remained constant for drought treated samples. Relative transcript level of *T-SAND* varied in all samples but maximum level was observed in heat treatment. From these results it can be concluded that expression level of most of the genes is not constant, it varies with different treatments. So there is need to analyze the expression stability of each gene.

### Expression stability analysis

#### GeNorm analysis

**Fig 3:**
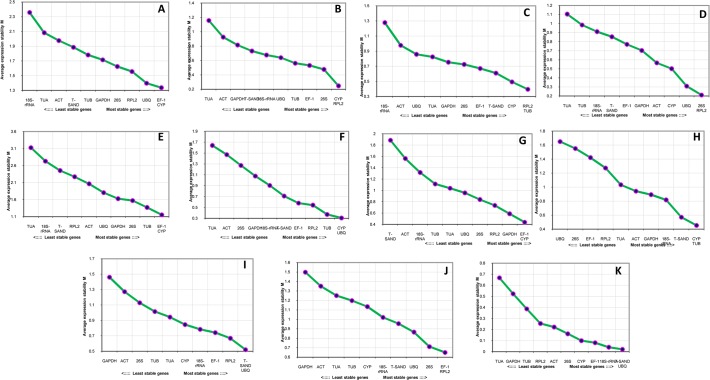
Expression stability and ranking of the candidate reference genes by geNorm. Expression stability and ranking of 11 reference genes calculated with geNorm in all the samples (A), Wounding (B), Salt (C), Drought (D), Heat (E), Cold (F), Tissue (G), MJ (H), SA (I), ABA (J) and Biotic (K). A lower M value indicates more stable gene expression.

The expression stability of candidate reference genes under different stress conditions was evaluated by using different approaches. Relative quantities of all samples were exported to geNorm (version 3.5) to analyze gene expression stability. Stability analysis by geNorm is based on the M value, which is calculated by stepwise exclusion of the unstable genes followed by recalculation of M values [27]. M value less than the threshold value of 1.5 has been recommended by Vandesompele et al [27]. In our analysis only 3 genes showed M value under the recommended value of 1.5 and rest of the five genes were above this value. It was observed that the stability of the genes varied according with different treatments and most of them showed inconsistently under all experimental conditions. Starting from the two most stable genes on the right (lowest M value), the genes are ranked according to decreasing expression stability, ending with the most unstable genes on the left with highest M value. The most stable reference genes were not identical in all individual stress treated samples. *TUB* and *RPL2* (0.39) seems to be the most stable genes in salt treated samples, meanwhile *RPL2* and *26S* (0.21) in drought treatment, and *CYP* and *EF-1* (1.15) in heat treated samples. Similarly, among other 11 genes *CYP* and *UBQ* were found to be most stable genes with M value of 0.30 for wounding and cold treatment. *CYP*, *TUB* (0.45) for MJ samples and *T-SAND*, *UBQ* (0.52) for SA treated samples showed lowest M values, thus were most stable genes. For ABA treatment *RPL2* and *EF-1* (0.64) exhibited stable expression as compare to other genes. Under biotic stress *UBQ* and *T-SAND* genes were on the top with lowest M value of 0.021. For all combined treatments, different tissues and heat treatment *CYP* and *EF-1* genes were found to be most stable (Fig. 3). Rest of the genes were found to be unstable with higher M values than cut off value of 1.5. Among all the reference genes examined *18S-rRNA* and *TUA* were found to be the least stable with highest M value.

**Fig 4:**
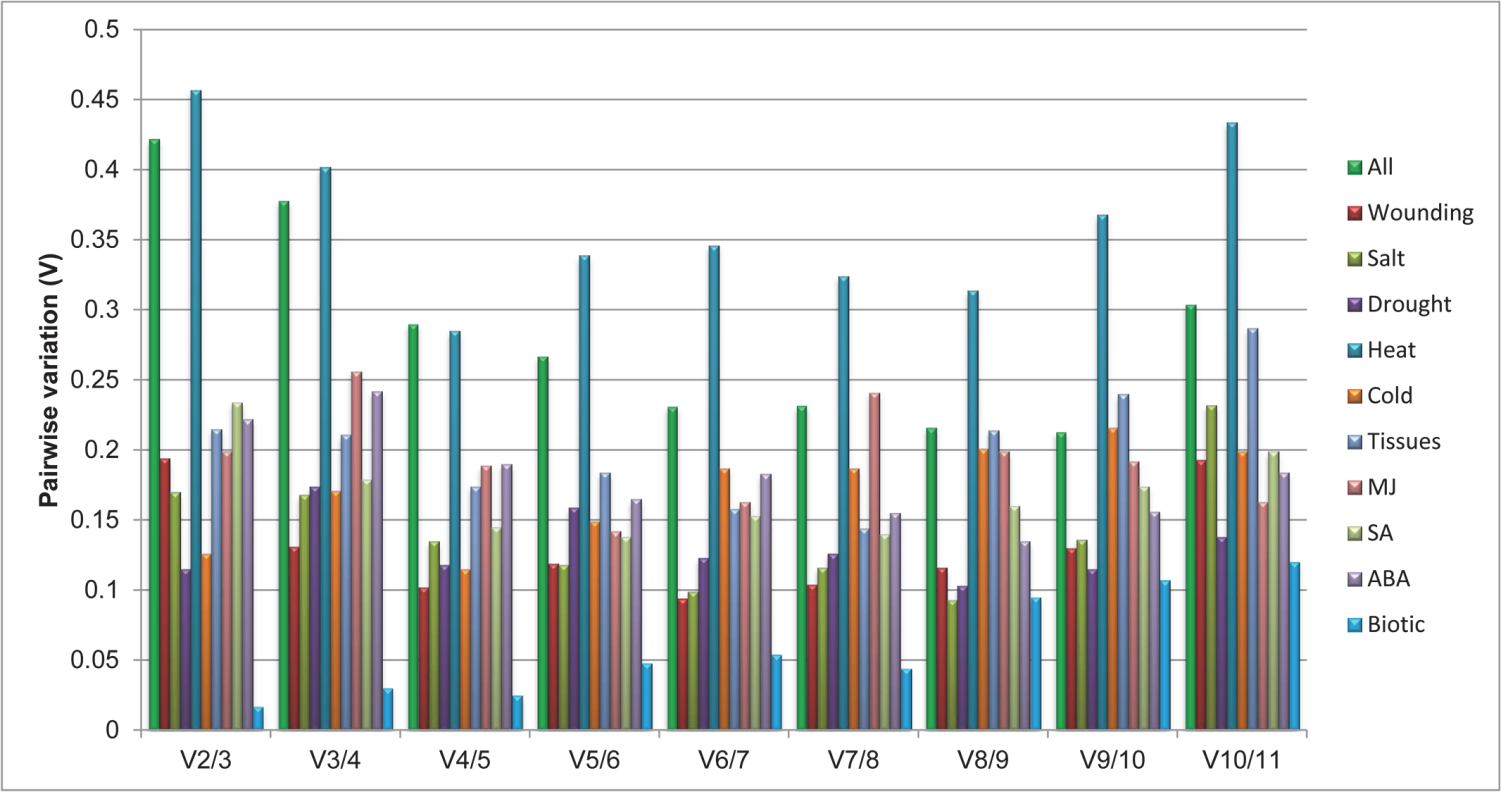
Pairwise variation calculated by geNorm to determine the minimum number of reference genes required for accurate normalization in each experimental set. The cut off value is 0.15, below which the inclusion of an additional reference gene is not required.

To find out the optimal number of reference genes in each experimental condition, geNorm also calculate pairwise variation (V). Vandesompele and coworkers [27] usually recommends 0.15 as a cutoff value to determine the optimal number of reference genes. The inclusion of additional gene if V value is below this cutoff value is not considered. However, 0.15 is not an absolute cutoff value but rather an ideal value depending on the amount of genes and type of samples tested [32, 33]. Pairwise variation analysis showed that the optimal number of reference genes may be different for distinct stress samples. Analysis of the pairwise variation in cold, drought and biotic treatments revealed that the Vn+1 value is less than cutoff value of 0.15, indicating that only two reference genes would be sufficient for normalizing gene expression in these stress treatment (Fig. 4). For wounding treatment pairwise variation at the V2/3 value was 0.194, which is above the threshold of 0.15, and with the addition of next gene it drops down to stable value of 0.131, indicating that the proper normalization require at least three reference genes. Therefore, *CYP*, *RPL2*, and *26S* can be used as reference genes to normalize gene expression data in wounding treated samples. With heat treated samples all genes pairwise variation or V number always remained above cutoff value of 0.15. On the other hand, four genes for SA and five genes for the MJ treated samples are required for the normalization. Among different tissue subset addition of seventh gene lower the V value below cutoff. Similarly addition of *TUB* (eighth gene) lower the V value in case of ABA treated samples. For all the samples taken together for analysis, it was observed that there was not a significant difference in the V numbers, but the instability was increased with the addition of *TUA* (V9/10) and *18S-rRNA* (V10/11).

#### NormFinder analysis

The Expression stability of 11 candidate genes was further analyzed using MS Excel-based NormFinder software. NormFinder measures gene expression stability by comparing the variation within and between user-defined sample groups [28]. Candidate control genes with lowest stability values have the minimum intra and intergroup variation and thus are top ranked. For each candidate gene, NormFinder provides a stability value (SV) that is a direct measurement of expression variation. To perform NormFinder analysis log transformed data was used as input and further divided into 2 types of subgroups. First subgroup consists of ten divisions based on different treatment and other group includes only four subgroups (biotic, abiotic treatments, different tissues and plant growth regulators,). At the same time, all samples with no subgroups were also analyzed using this approach as well. Division of samples into subgroups did not showed significant effect on the results obtained by NormFinder. With ten subgroups and without any subgroups NormFinder identified *CYP* as the most stable gene with lowest SV values for all treatments (Table 2) which was consistent with the result given by geNorm. Within ten subgroups *CYP* and *EF-1* was found to be the best combination of genes across all samples with a stability value of 0.419, which is lower than the single best *CYP* (0.543). If samples categorized into four subgroups again *CYP* and EF-1 (0.129) combination was found to be best for all treatments. For individual treatments *CYP* for wounding, different tissues and MJ treated samples, *T-SAND* for salt and SA treatment, *26S* for heat and drought treated samples were identified to be most reliable genes. Similarly, *TUB* for cold, *UBQ* for biotic stress, and *18s-rRNA* for ABA treatment were on top position. Overall analysis *CYP*, *26S*, and *T-SAND* showed a remarkable stability of their expression levels and were always classified among the top positions while *TUA*, *18s-rRNA* and *GAPDH* were always included among the least stable reference genes.

**Table 2:** Ranking orders of the candidate reference genes according to their stability value calculated NormFinder algorithm.

Rank	No Groups	Ten Groups	Four Groups	Wounding	Salt	Drought	Heat	Cold	Tissue	MJ	SA	ABA	Biotic
1	*CYP*	*CYP*	*CYP*	*CYP*	*T-SAND*	*26S*	*26S*	*TUB*	*CYP*	*CYP*	*T-SAND*	*18S-rRNA*	*UBQ*
**SV**	0.478	0.543	0.380	0.174	0.191	0.073	0.674	0.060	0.152	0.154	0.101	0.284	0.007
**2**	*EF-1*	*EF-1*	*EF-1*	*RPL2*	*CYP*	*UBQ*	*TUB*	*RPL2*	*RPL2*	*T-SAND*	*CYP*	*T-SAND*	*T-SAND*
**SV**	0.729	0.679	0.456	0.236	0.287	0.108	0.712	0.289	0.323	0.174	0.180	0.361	0.007
**3**	*UBQ*	*UBQ*	*UBQ*	*TUB*	*GAPDH*	*RPL2*	*CYP*	*EF-1*	*GAPDH*	*TUB*	*18S-rRNA*	*TUB*	*EF-1*
**SV**	0.855	0.704	0.551	0.238	0.424	0.160	0.778	0.322	0.382	0.541	0.375	0.566	0.013
**4**	*RPL2*	*RPL2*	*26S*	*18S-rRNA*	*TUB*	*ACT*	*GAPDH*	*T-SAND*	*EF-1*	*TUA*	*RPL2*	*CYP*	*CYP*
**SV**	0.902	0.749	0.651	0.271	0.431	0.386	1.086	0.346	0.412	0.562	0.471	0.625	0.013
**5**	*TUB*	*T-SAND*	*RPL2*	*T-SAND*	*TUA*	*CYP*	*EF-1*	*UBQ*	*26S*	*ACT*	*TUA*	*TUA*	*18S-rRNA*
**SV**	1.016	0.798	0.735	0.414	0.433	0.390	1.104	0.394	0.631	0.719	0.527	0.681	0.014
**6**	*GAPDH*	*TUB*	*GAPDH*	*UBQ*	*EF-1*	*GAPDH*	*ACT*	*CYP*	*TUA*	*RPL2*	*TUB*	*EF-1*	*26S*
**SV**	1.026	0.798	0.760	0.428	0.459	0.621	1.112	0.494	0.862	0.926	0.587	0.683	0.132
**7**	*26S*	*26S*	*T-SAND*	*EF-1*	*RPL2*	*EF-1*	*UBQ*	*18S-rRNA*	*TUB*	*18S-rRNA*	*UBQ*	*UBQ*	*ACT*
**SV**	1.045	0.845	1.069	0.540	0.473	0.640	1.471	0.943	0.870	0.998	0.605	0.693	0.261
**8**	*T-SAND*	*GAPDH*	*TUB*	*26S*	*UBQ*	*T-SAND*	*RPL2*	*GAPDH*	*UBQ*	*EF-1*	*EF-1*	*RPL2*	*RPL2*
**SV**	1.148	0.914	1.092	0.563	0.552	0.661	1.491	0.955	0.904	1.035	0.711	0.705	0.286
**9**	*ACT*	*TUA*	*TUA*	*ACT*	*26S*	*18S-rRNA*	*T-SAND*	*ACT*	*18S-rRNA*	*GAPDH*	*ACT*	*ACT*	*TUB*
**SV**	1.283	1.024	1.151	0.765	0.567	0.743	1.875	1.346	1.308	1.162	1.117	0.990	0.553
**10**	*TUA*	*ACT*	*ACT*	*GAPDH*	*ACT*	*TUB*	*18S-rRNA*	*26S*	*ACT*	*UBQ*	*26S*	*26S*	*GAPDH*
**SV**	1.545	1.205	1.370	0.806	0.973	0.757	2.801	1.448	1.504	1.220	1.189	0.999	0.817
**11**	*18S-rRNA*	*18S-rRNA*	*18S-rRNA*	*TUA*	*18S-rRNA*	*TUA*	*TUA*	*TUA*	*T-SAND*	*26S*	*GAPDH*	*GAPDH*	*TUA*
**SV**	2.296	1.384	1.981	1.468	1.762	1.045	3.278	1.500	2.178	1.245	1.506	1.390	0.907

Note. Higher Stability value (SV) indicates genes with low transcriptional stability, whereas lower (SV) values indicate genes with high transcriptional stability.

#### BestKeeper analysis

BestKeeper, an Excel-based tool, estimated the coefficient of correlation (r) and BestKeeper Index (geometric mean) to find the most stably expressed genes [29]. Raw Cq values of each gene were used to calculate the coefficient of variance (CV) and the standard deviation (SD). The genes with the lowest value of coefficient of variance and standard deviation (CV±SD) are identified as the most stable reference gene. Genes that show a SD value less than 1 are considered acceptable [33–35]. In our study, *UBQ* (SD = 0.78) and *RPL2* (SD = 0.91) were found to have stable expression in all the samples, while *18S-rRNA* (SD = 2.06) showed least stable expression (Table 3). Further in individual treatments, BestKeeper analysis revealed that for, drought, ABA and all samples taken together *UBQ* emerged as the most stably expressed gene, while for heat, MJ and SA stress *T-SAND* was found to be most stably expressed gene. However, *EF-1* for salt, *RPL2* for wounding, *CYP* for different tissues, *18Sr-RNA* for cold and *TUB* for biotic stress had the lowest standard deviation, thus exhibit most stable expression.

**Table 3:** Ranking orders of the candidate reference genes according to their standard deviation calculated Bestkeeper algorithm.

Rank	Total	Wounding	Drought	Salt	Heat	Cold	Tissue	MJ	SA	ABA	Biotic
1	*UBQ*	*RPL2*	*UBQ*	*EF-1*	*T-SAND*	*18S-rRNA*	*CYP*	*T-SAND*	*T-SAND*	*UBQ*	*TUB*
**SD**	0.78	0.19	0.29	*0*.*36*	0.75	0.29	0.39	0.57	0.24	0.28	0.12
**2**	*RPL2*	*CYP*	*RPL2*	*GAPDH*	*ACT*	*TUB*	*EF-1*	*RPL2*	*UBQ*	*CYP*	*RPL2*
**SD**	0.91	0.22	0.34	0.42	*0*.*98*	0.66	0.44	0.67	0.32	0.52	0.14
**3**	*CYP*	*18S-rRNA*	*26S*	*T-SAND*	*RPL2*	*CYP*	*UBQ*	*EF-1*	*RPL2*	*EF-1*	*UBQ*
**SD**	1.04	0.35	0.35	0.54	1	0.7	0.52	0.7	0.44	0.56	0.16
**4**	*EF-1*	*26S*	*CYP*	*26S*	*18S-rRNA*	*UBQ*	*26S*	*CYP*	*EF-1*	*T-SAND*	*EF-1*
**SD**	1.09	0.43	0.45	0.6	1.3	0.75	0.56	0.78	0.45	0.82	0.23
**5**	*T-SAND*	*UBQ*	*18S-rRNA*	*TUB*	*26S*	*RPL2*	*GAPDH*	*UBQ*	*18S-rRNA*	*TUB*	*18S-rRNA*
**SD**	1.29	0.45	0.46	0.73	1.72	0.92	0.68	0.8	0.59	0.97	0.25
**6**	*26S*	*T-SAND*	*EF-1*	*CYP*	*GAPDH*	*T-SAND*	*TUB*	*TUB*	*CYP*	*26S*	*ACT*
**SD**	1.37	0.48	0.47	0.83	2.27	0.95	0.85	0.99	0.69	0.99	0.32
**7**	*TUB*	*TUB*	*GAPDH*	*RPL2*	*CYP*	*EF-1*	*RPL2*	*26S*	*26S*	*RPL2*	*T-SAND*
**SD**	1.48	0.53	0.59	0.93	2.35	0.96	0.93	1.06	0.71	1.01	0.38
**8**	*GAPDH*	*EF-1*	*T-SAND*	*TUA*	*TUB*	*26S*	*TUA*	*TUA*	*TUA*	*18S-rRNA*	*TUA*
**SD**	1.62	0.65	0.63	1	2.51	1.35	0.94	1.08	0.89	1.03	0.48
**9**	*ACT*	*ACT*	*ACT*	*UBQ*	*UBQ*	*ACT*	*18S-rRNA*	*18S-rRNA*	*TUB*	*TUA*	*26S*
**SD**	1.9	*0*.*96*	*0*.*69*	1.04	2.55	*1*.*7*	1.73	1.39	1.04	1.39	0.5
**10**	*TUA*	*GAPDH*	*TUB*	*ACT*	*EF-1*	*GAPDH*	*ACT*	*ACT*	*ACT*	*ACT*	*CYP*
**SD**	1.91	1.02	1.04	1.36	2.65	1.71	1.84	1.43	1.73	1.83	0.64
**11**	*18S-rRNA*	*TUA*	*TUA*	*18S-rRNA*	*TUA*	*TUA*	*T-SAND*	*GAPDH*	*GAPDH*	*GAPDH*	*GAPDH*
**SD**	2.06	1.56	1.45	1.61	4.45	2.09	2.65	1.53	1.8	1.9	0.97

Note. Reference genes with SD values greater than 1 are considered as inconsistent and should be excluded.

Further, to evaluate the results obtained from the three software, geNorm, NormFinder and Bestkeeper, we used comparative ΔCt. The comparative ΔCt method ranks the reference genes based on their mean standard deviation in the pairwise comparisons [30]. For comprehensive ranking of candidate genes average Cq values were used as such into the program and GM was calculated. The stability rankings results obtained from ΔCt method showed high similarity with the results described by four softwares geNorm, NormFinder and Bestkeeper (Table 4). Further, RefFinder was used to compare different algorithms for the selection of the top four most stable and two least stable reference genes (Table 5). RefFinder (http://www.leonxie.com/referencegene.php), a web-based comprehensive tool which uses all these four algorithms geNorm, NormFinder, BestKeeper and comparative ΔCt methods, and assigns an appropriate weight to an individual gene and calculates the geometric mean for comprehensive ranking of all reference genes.

**Table 4:** Ranking orders of the candidate reference genes according to their average standard deviation calculated ΔCt method.

Rank	All	Wounding	Salt	Drought	Heat	Cold	Tissue	MJ	SA	ABA	Biotic
**1**	*CYP*	*CYP*	*T-SAND*	*26S*	*CYP*	*TUB*	*CYP*	*CYP*	*T-SAND*	*18S-rRNA*	*EF-1*
**Aver. STDEV**	1.87	0.81	0.93	0.79	2.5	1.19	1.31	1.23	1.07	1.21	0.44
**2**	*EF-1*	*RPL2*	*CYP*	*UBQ*	*TUB*	*RPL2*	*EF-1*	*T-SAND*	*CYP*	*T-SAND*	*CYP*
**Aver. STDEV**	1.98	0.86	0.98	0.82	2.55	1.24	1.43	1.25	1.14	1.24	0.44
**3**	*UBQ*	*TUB*	*TUB*	*RPL2*	*26S*	*EF-1*	*GAPDH*	*TUB*	*18S-rRNA*	*TUB*	*T-SAND*
**Aver. STDEV**	2.1	0.88	1.05	0.84	2.57	1.24	1.45	1.36	1.22	1.36	0.45
**4**	*RPL2*	*18S-rRNA*	*RPL2*	*CYP*	*EF-1*	*UBQ*	*RPL2*	*TUA*	*RPL2*	*EF-1*	*UBQ*
**Aver. STDEV**	2.15	0.94	1.07	0.97	2.71	1.26	1.5	1.47	1.23	1.37	0.46
**5**	*TUB*	*EF-1*	*GAPDH*	*ACT*	*GAPDH*	*CYP*	*26S*	*ACT*	*UBQ*	*RPL2*	*18S-rRNA*
**Aver. STDEV**	2.19	0.97	1.12	0.98	2.77	1.33	1.58	1.54	1.32	1.41	0.48
**6**	*GAPDH*	*26S*	*EF-1*	*GAPDH*	*ACT*	*T-SAND*	*UBQ*	*18S-rRNA*	*TUA*	*UBQ*	*26S*
**Aver. STDEV**	2.24	0.98	1.12	1.14	2.8	1.35	1.74	1.7	1.32	1.42	0.5
**7**	*26S*	*UBQ*	*TUA*	*EF-1*	*UBQ*	*18S-rRNA*	*TUA*	*RPL2*	*TUB*	*CYP*	*ACT*
**Aver. STDEV**	2.26	1.02	1.15	1.16	3.03	1.77	1.78	1.74	1.36	1.42	0.57
**8**	*T-SAND*	*T-SAND*	*26S*	*T-SAND*	*RPL2*	*GAPDH*	*TUB*	*GAPDH*	*EF-1*	*TUA*	*RPL2*
**Aver. STDEV**	2.37	1.09	1.18	1.21	3.04	1.8	1.79	1.86	1.38	1.46	0.59
**9**	*ACT*	*GAPDH*	*UBQ*	*18S-rRNA*	*T-SAND*	*ACT*	*18S-rRNA*	*EF-1*	*26S*	*26S*	*TUB*
**Aver. STDEV**	2.47	1.27	1.28	1.29	3.28	2.18	2.28	1.86	1.85	1.67	0.91
**10**	*TUA*	*ACT*	*ACT*	*TUB*	*18S-rRNA*	*26S*	*ACT*	*26S*	*ACT*	*ACT*	*GAPDH*
**Aver. STDEV**	2.71	1.37	1.62	1.32	4.25	2.29	2.57	2.06	1.87	1.76	1.19
**11**	*18S-rRNA*	*TUA*	*18S-rRNA*	*TUA*	*TUA*	*TUA*	*T-SAND*	*UBQ*	*GAPDH*	*GAPDH*	*TUA*
**Aver. STDEV**	3.61	2.29	2.62	1.64	4.91	2.39	3.34	2.09	2.31	2.17	1.32

Note. Higher Aver. STDEV values indicate genes with low transcriptional stability, whereas lower Aver. STDEV values indicate genes with high transcriptional stability.

**Table 5:** Relative stability ranking of potential reference genes based on geNorm, NormFinder, Bestkeeper and ΔCt method.

Experimental conditions	Four most stable genes				Recommended comprehensive ranking	Two Least stable genes
	geNorm	NormFinder	Bestkeeper	ΔCt		
**All**	*CYP*,*EF-1*, *UBQ*,*RPL2*	*TUB*, *CYP*, *UBQ*, *26S*	*UBQ*, *RPL2*, *CYP*,*EF-1*	*CYP*, EF-1, UBQ, RPL2	*CYP*, *EF-1*,*UBQ*,*RPL2*	*18S-rRNA*, *TUA*
**Wounding**	*CYP*, *UBQ*, *TUB*, *RPL2*	*CYP*, *RPL2*, *TUB*, *18S-rRNA*	*RPL2*, *CYP*, *18S-rRNA*, *26S*	*CYP*, *RPL2*, *TUB*, *18S-rRNA*	*CYP*, *RPL2*, *TUB*, *18S-rRNA*	*TUA*, *GAPDH*
**Salt**	*TUB*, *RPL2*, *CYP*,*T-SAND*	*UBQ*, *GAPDH*, *CYP*, *EF-1*	*EF-1*,*GAPDH*,*T-SAND*, 26S	*T-SAND*, *CYP*,*TUB*,*RPL2*	*T-SAND*,*TUB*, *CYP*,*EF-1*	*18S-rRNA*, *ACT*
**Drought**	*26S*, *RPL2*, *UBQ*, *CYP*	*26S*, *UBQ*, *RPL2*, *ACT*	*UBQ*, *RPL2*, *26S*, *CYP*	*26S*, *UBQ*, *RPL2*, *CYP*	*26S*, *UBQ*, *RPL2*, *CYP*	*TUA*, *TUB*
**Heat**	*CYP*, *EF-1*, *TUB*, *26S*	*26S*, *TUB*, *CYP*, *GAPDH*	*T-SAND*, *ACT*, *RPL2*, *18S-rRNA*	*CYP*, *TUB*, *26S*, *EF-1*	*CYP*, *26S*, *TUB*, *EF-1*	*TUA*, *18S-rRNA*
**Cold**	*CYP*, *UBQ*, *TUB*, *RPL2*	*TUB*, *RPL2*, *EF-1*, *T-SAND*	*18S-rRNA*, *TUB*, *CYP*, *UBQ*	*TUB*, *RPL2*, *EF-1*, *UBQ*	*TUB*, *UBQ*, *RPL2*, *CYP*	*TUA*, *ACT*
**Tissue**	*CYP*, *EF-1*, *GAPDH*,*RPL2*	*CYP*, *RPL2 GAPDH*, *EF-1*	*CYP*, *EF-1*, *UBQ*, *26S*	*CYP*,*EF-1*,*GAPDH*, *RPL2*	*CYP*,*EF-1*,*GAPDH*,*RPL2*	*T-SAND*, *ACT*
**MJ**	*CYP*, *TUB*, *T-SAND*, *18S-rRNA*	*CYP*, *T-SAND*, *TUB*, *TUA*	*T-SAND*, *RPL2*, *EF-1*, *CYP*	*CYP*, *T-SAND*, *TUB*, *TUA*	*CYP*, *T-SAND*, *TUB*, *RPL2*	*26S*, *UBQ*
**SA**	*T-SAND*, *UBQ*, *RPL2*, *EF-1*	*T-SAND*, *CYP*, *18S-rRNA*, *RPL2*	*T-SAND*, *UBQ*, *RPL2*, *EF-1*	*T-SAND*, *CYP*, *18S-rRNA*, *RPL2*	*T-SAND*, *UBQ*, *RPL2*, *CYP*	*GAPDH*, *ACT*
**ABA**	*RPL2*, *EF-1*, *26S*, *UBQ*	*18S-rRNA*, *T-SAND*, *TUB*, *CYP*	*UBQ*, *CYP*, *EF-1*, *T-SAND*	*18S-rRNA*, *T-SAND*, *TUB*, *EF-1*	*18S-rRNA*, *EF-1*, *T-SAND*, *UBQ*	*GAPDH*, *ACT*
**Biotic**	*UBQ*, T-SAND, *EF-1*, *18S-rRNA*	*UBQ*, T-SAND, *EF-1*,*CYP*	*TUB*, *RPL2*,*UBQ*,*EF-1*	*EF-1*, *CYP*, *T-SAND*, *UBQ*	*UBQ*, *T-SAND*, *EF-1*, *TUB*	*GAPDH*, *TUA*

#### Validation of reference genes

**Fig 5:**
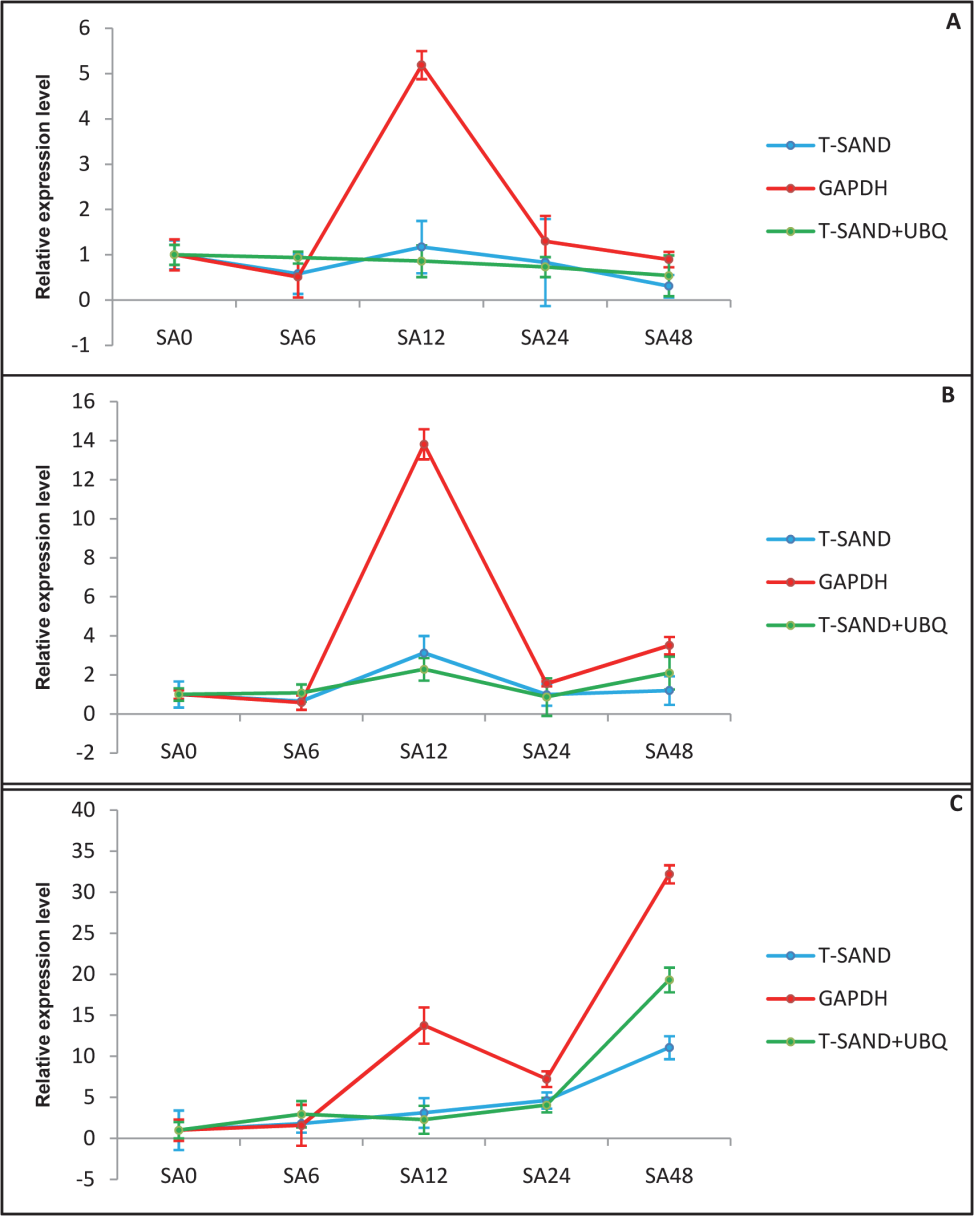
Relative expression level of withanolides biosynthesis genes in response to SA treatment. Transcript level relative quantification of HMGR (A), CAS (B) and P450 (C). The most stable genes recommended for SA treatment (*T-SAND* and UBQ) and the least stable gene GAPDH were used for normalization. For *T-SAND* and UBQ geometric mean was calculated and used for normalization of expression. Error bars show standard deviation calculated from three biological replicates.

In order to further evaluate the expression stability of selected reference genes, we analyzed the expression of three genes that play key role in withanolide biosynthesis pathway. The expression of *HMGR* and *CAS* was not significantly affected by treatment with SA. The results reveal that the expression level of *HMGR* was highest after 12 h of treatment and then decline thereafter. The relative expression of *CAS* showed a variable pattern of expression where it showed significant upregulation after 12 and 48 h of treatment. Expression analysis of *P450* showed upregulation after SA treatment and reached maximum after 48 h (Fig. 5).

## Discussion

The qRT-PCR is one of the most frequently used method for accurate and sensitive quantification of gene expression analysis. However, these outcomes are strongly dependent on reliable reference controls [26]. Although a single endogenous control for normalization is often used, it is largely limited by stability of gene expression across different experimental condition. Thus, it was considered essential to explore the expression stability of the control genes and to find out ideal endogenous reference genes with stable gene expression under particular set of conditions. In order to screen such reliable reference genes, we analyzed expression profiles of 11 genes under different experimental conditions and different software packages: geNorm, NormFinder, Bestkeeper and comparative ΔCt method. Recently, these statistical algorithms have been used to validate and identify the best reference genes for qRT-PCR data normalization across a variety of tissues, organs, developmental stages, and stress conditions in various plant species like peanut [36], tomato [22], barley, sorghum, wheat, maize [37].

As shown in box-and-whiskers plots, the selected genes exhibited a relatively wide range of expression that confirmed that no single gene had a constant gene expression under different stress conditions. When the outcome of these four algorithms was compared, for wounding, salt, drought, MJ, SA, biotic stress and for all samples together we found highly consistent results for first position, with slight changes in other positions. For example, for wounding and MJ treatment, all algorithms choose *CYP* on top, except Bestkeeper which ranked *CYP* at 2^nd^ position. From these results, *CYP* was found to be appropriate gene for wounding and MJ treated samples, and this is consistent with the expression stability of the other ribosomal protein *RPL2* which is a widely used gene for normalization in expression studies [38]. Three algorithms geNorm, NormFinder and ΔCt method selected *26S and UBQ* as best genes for drought treated samples and recommended as best genes for normalization. Previous studies in rice showed the highest expression stability of *UBQ5* and *EF-1α* among 10 reference genes tested [39]. Similarly, UBQ was reported as most stably expressed gene in *Platycladus orientalis* for salt stress samples [34]. *T-SAND* for SA treatment and *CYP* among different tissues were found to be the best reference genes for normalization by all methods. Previous studies in Buckwheat also identified *T-SAND* as one of the top-ranked reference gene [40]. *18S-rRNA* was identified as the most stable gene for ABA treated samples by NormFinder and ΔCt method. For heat and cold treated samples all software packages showed high variation for ideal genes. GeNorm and ΔCt method ranked *CYP* as common best genes for heat treatment but according to NormFinder and Bestkeeper *26S* and *T-SAND* was most stable gene, respectively. The TUB gene plays an important role in cell structure maintenance. In our study for cold treated samples, *TUB* was found to be most stable when analyzed by NormFinder and ΔCt method but geNorm ranked *TUB* on 3^rd^ position, and Bestkeeper on 2^nd^ position. We observed that the top three positions of reference genes for all samples together were almost the same when determined by geNorm, NormFinder and comparative ΔCt and not by Bestkeeper. Because all these software packages are based on different algorithms, this variation in the results can be expected. Further, these results clearly indicated that it is extremely important to validate the expression stability of these genes prior to use for normalization because none of the selected genes showed constant gene expression in all the tested samples.

*CYP* was found to be the best gene for wounding, heat, MJ, biotic stress, different tissues and combined stress samples together. Our results are consistent with results found by Jain et al 2008 [41] who also reported that according to geNorm and ΔCt analysis, eukaryotic elongation factor 1-beta (*ELF1B*) and cyclophilin (*CYP*2) could be used as reference genes to normalize gene expression in soybean. Earlier, CYP was also identified as most stable gene for normalization in Siberian Apricot Germplasms [42] and *Petunia hybrida* [43].

To date, actin *(ACT)* has been used most frequently as the internal reference gene in gene expression analysis by qRT-PCR and semi-quantitative PCR in *W*. *somnifera* [18, 24, 44] and this genes was selected without any validation study to evaluate its suitability under different experimental conditions. However, various studies indicate that under different experimental conditions the expression pattern of commonly used housekeeping genes can vary considerably [45–47]. Further, this may not be true in all plant species like actin gene exhibit inappropriate expression stability to use as a reference gene in one of grass species, *Brachypodium distachyon* [32]. Further, in rice, the expression analysis revealed that *ACT* was not even detected as expressed, in one or more tissue/stress microarray experiments [48]. In another reference gene validation study in peanut, *ACT* gene was found to be stable only in vegetative stages not in biotic and abiotic stress treatments, which indicates the influence of the experimental conditions on its expression [36].

For Overall analysis RefFinder was used in which 4 statistical approaches were equally weighted and combined to find out four top ranked genes (Table 5). And these results suggested that each tested experimental stress condition needs a specific set of reference genes for normalization. But all these methods found that *TUA*, *GAPDH* and *18S-rRNA* were the most unstable genes, thus suggested inappropriate as the reference genes for gene expression analysis through qRT-PCR analysis in *W*. *somnifera*. Due to high expression variability of *TUA* it was also not considered as suitable internal controls in different verities and organs of litchi [49]. On the other hand *GAPDH* was considered as the most stable gene for normalization in different coffee cultivars and drought stress [50]. *GAPDH* is a key enzyme involved in glycolysis but from our results it seems that it may also be involved in other cellular functions as well. In earlier studies, *18S-rRNA* was used as an internal reference gene and it was identified as the most reliable reference gene for normalization in rice [51] and cereals [52]. However in *W*. *Somnifera* expression of *18S-rRNA* was unstable for most of the treated samples and not recommended as a reliable reference gene for normalization. These results suggest that reference genes are not only implicated in the basal cell metabolism but also may participate in other cellular functions and are regulated differently in different plant species [20].

For validation studies we used the two most stable genes (*T-SAND* and *UBQ*) and one least stable gene (*GAPDH*). In recent years, intensive research has been carried out on salicylic acid (SA) due to its function as an endogenous signal which plays a important role during the plant response to biotic and abiotic stresses such as drought, chilling, heavy metal toxicity, heat, and osmotic stress [53]. SA also importantly contributes to growth and development regulation. Treatment with this signalling molecule has been reported to enhance secondary metabolites production in plants [54, 55]. Hence we choose SA to study its effect on the expression of withanolides biosynthetic pathway genes. *HMGR* catalyzes the irreversible conversion of 3-hydroxy-3-methylglutaryl coenzyme A (HMG-CoA) into mevalonic acid which is the key regulatory step of isoprenogenesis leading to the synthesis of IPP and DMAPP [56]. Our results showed that the expression of *HMGR* increased non-significantly with SA treatment, which matches the result obtained by Akhtar et al [57] which also showed slight upregulation after 24hrs of treatment followed by decline. The other important gene of withanolide biosynthetic pathway is *CAS* that leads to the formation of cycloartenol, which act as precursor to withanolides and diverse triterpenoids. There are no reports in literature about expression of *CAS* under SA treatment. Only one report is available which described the non significant effect of methyl jasmonate on *CAS* gene expression [58]. Recent studies in *W*. *somnifera* have shown that *P450* is an important gene of withanolides biosynthesis and expression of *P450* gene is significantly upregulated in *Withania* under SA treatments [18]. Our study also showed that the expression of *P450* gene was upregulated after SA treatment using *T-SAND* and *UBQ* as endogenous controls. The expression patterns of these three genes showed almost similar trends when most stable *T-SAND* and *UBQ* reference genes were used either singly or in combination. On the other hand when most unstable *GAPDH* was used as reference gene, expression was over estimated after 12 h and 48 h of SA treatment. This further indicated that using an unstable reference gene generated biases that could lead to misinterpretation of gene expression results and this gene is not appropriate for gene expression studies in *Withania*.

In conclusion, the present work provides useful background information about various reference genes selection for qRT-PCR studies in *W*. *somnifera*. The study will contribute significantly in background of upsurge the interest in genomics and transcriptomics studies in this high value medicinal plant.

## Supporting Information

S1 Fig RNA sample on a 2% agarose gel stained with ethidium bromide. (TIF)

S2 Fig PCR results of candidate reference genes. PCR products were run on a 2% agarose gel. Amplicons were of the expected sizes (*CYP*-87bp, *T-SAND*-90bp, *EF-1-*107bp, *GAPDH*-99bp, *TUB*-102bp, *UBQ*-111bp, *26S*-103bp, *18S-rRNA*-106bp, *TUA*-101bp, *RPL2-*119bp, *ACT*-116bp), L = 100bp ladder.
(TIF)

S3 Fig Dissociation curves for 11 potential reference genes and three validation genes: (A) *EF-1*, (B) *GAPDH*, (C) *CYP*, (D) *T-SAND*, (E) *26S*, (F) *TUA*, (G) *18S-rRNA*, (H) *RPL2*, (I) *TUB*, (J) *UBQ*, (K) *ACT*, (L) CAS, (M) HMGR, (N) P450. (TIF)

S1 Table Descriptions of withanolides biosynthetic pathway genes used for validation studies. (PDF)
