# Supplementary figures and images for: Evaluation and Selection of Candidate Reference Genes for Normalization of Quantitative RT-PCR in Withania somnifera (L.) Dunal
Source: PLoS One. 2015 Mar 13;10(3):e0118860. doi: 10.1371/journal.pone.0118860 (PMC4359125; doi:10.1371/journal.pone.0118860)

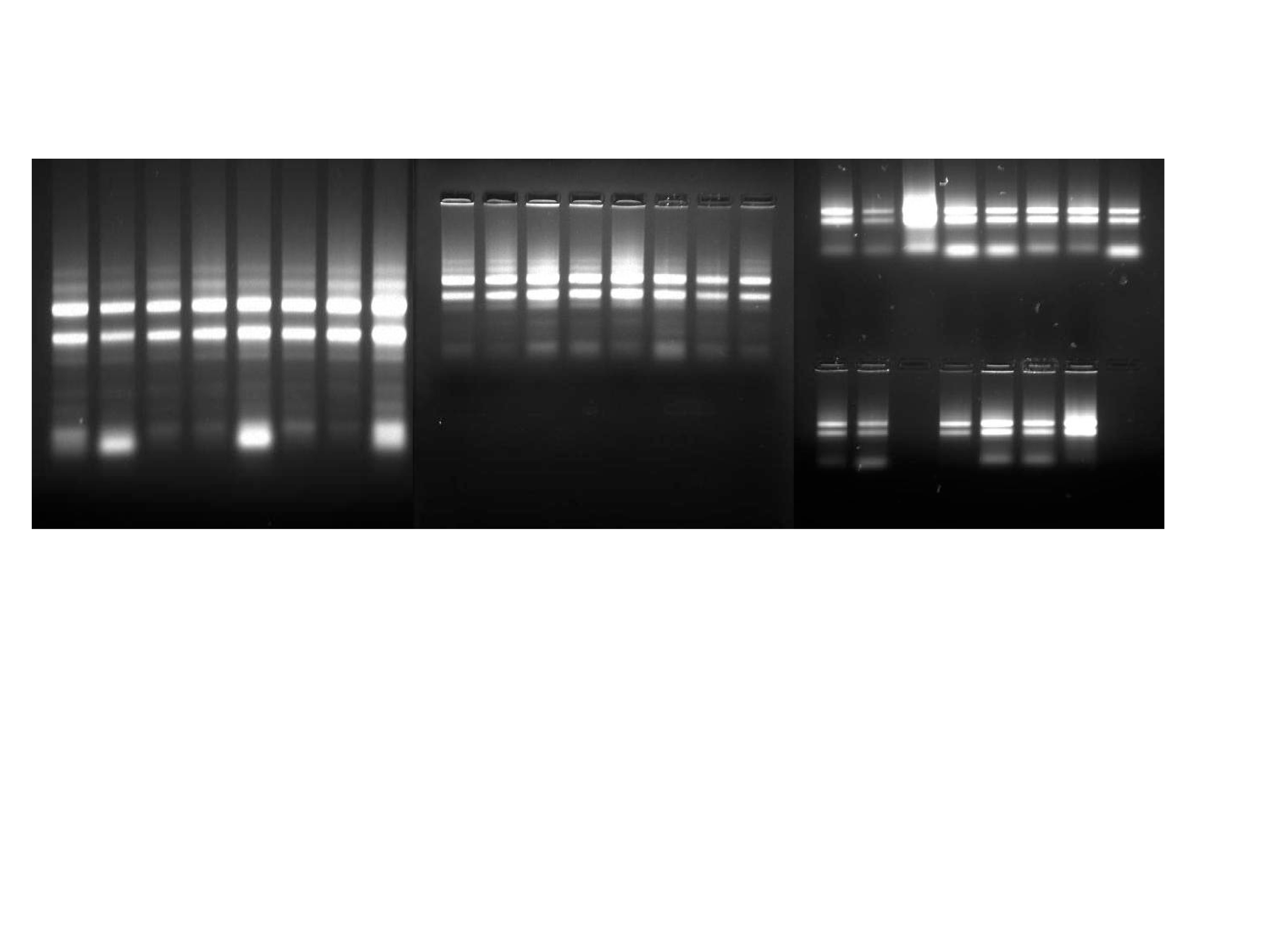

Supplement: S1 Fig — (TIF) [file pone.0118860.s001.tif]

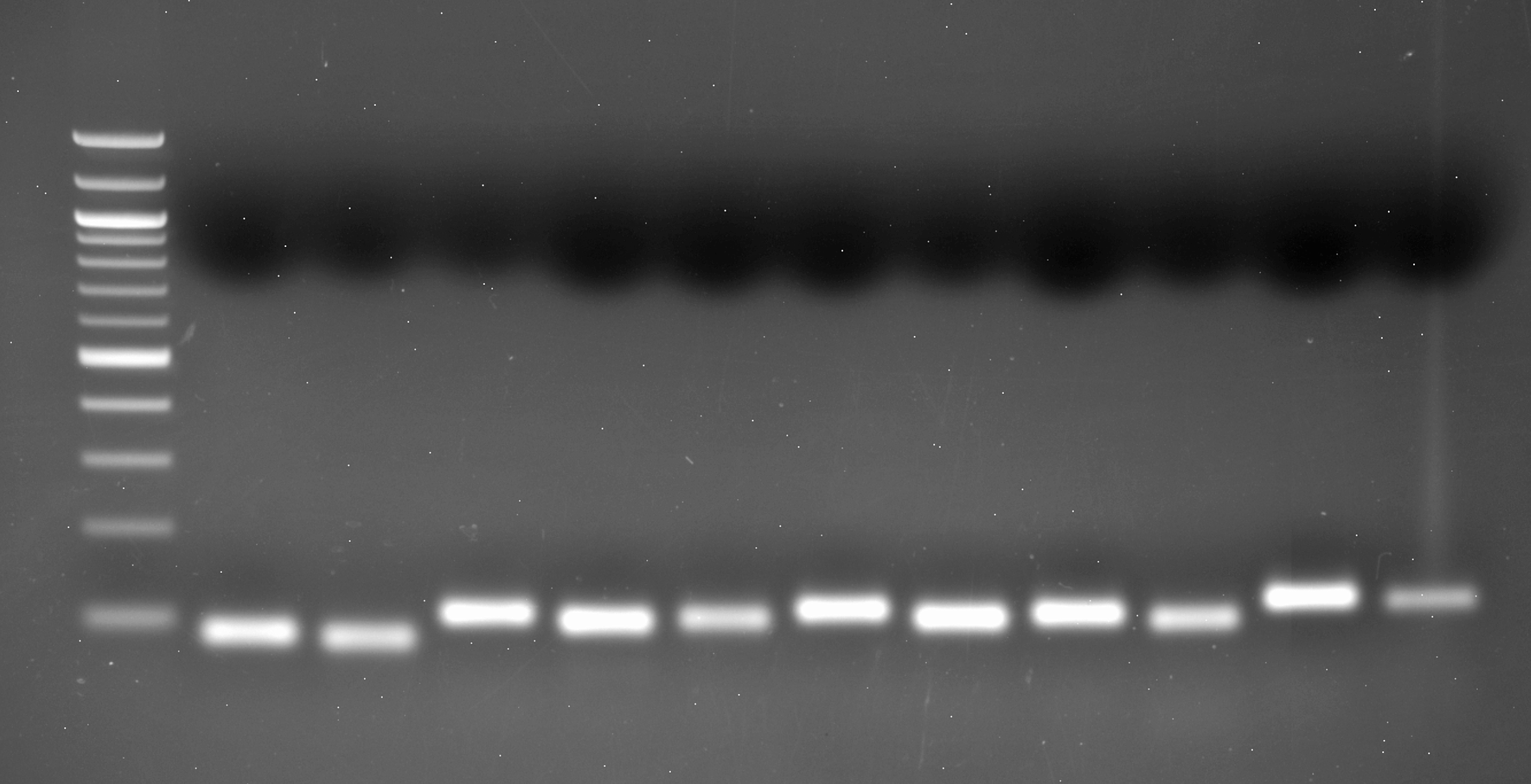

Supplement: S2 Fig — PCR products were run on a 2% agarose gel. Amplicons were of the expected sizes (CYP-87bp, T-SAND-90bp, EF-1-107bp, GAPDH-99bp, TUB-102bp, UBQ-111bp, 26S-103bp, 18S-rRNA-106bp, TUA-101bp, RPL2-119bp, ACT-116bp), L = 100bp ladder. (TIF) [file pone.0118860.s002.tif]

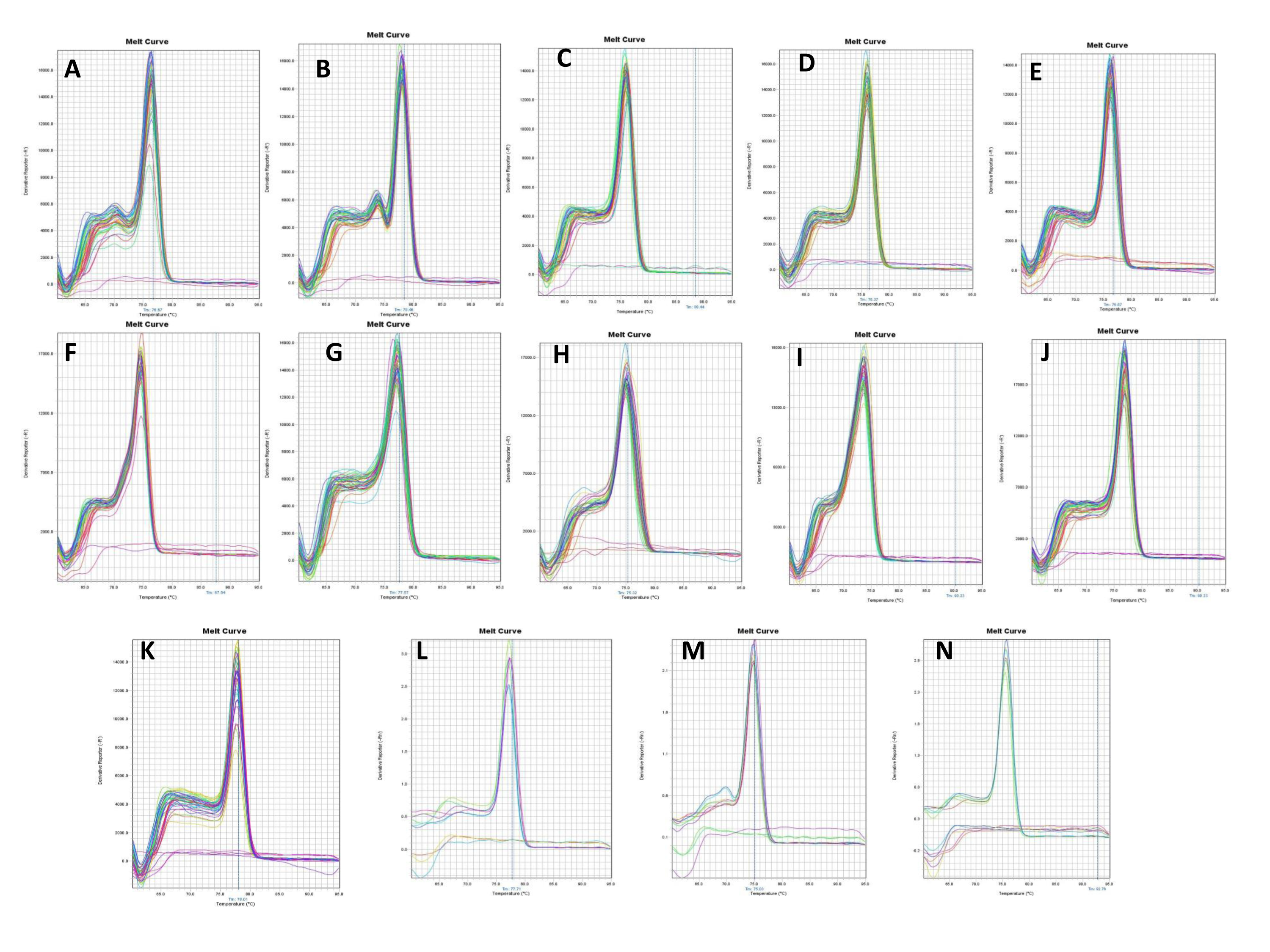

Supplement: S3 Fig — (TIF) [file pone.0118860.s003.tif]
