# Supplementary material for: Evaluation and Selection of Candidate Reference Genes for Normalization of Quantitative RT-PCR in Withania somnifera (L.) Dunal
Source: PLoS One. 2015 Mar 13;10(3):e0118860. doi: 10.1371/journal.pone.0118860 (PMC4359125; doi:10.1371/journal.pone.0118860)
Supplement: S1 Table — (PDF) [file pone.0118860.s004.pdf]

Gene Symble	Primer sequences (5' to 3') FP/RP	Gene description	Accession Number	Amplicon size (bp)	Tm (°C)
CAS	AACGCTTTGTCGGTCCTATC<br>CACACTCATTGCGAGCTTTATC	cycloartenol synthase	HM037907.1	105	62
HMGR	ATGATTTCCGGTGACGTAGTG<br>GCAATAGCAGAGCCAGTAAGA	3-hydroxy-3-methylglutaryl coenzyme A reductase	HQ293119.1	98	62
P450	ATGGTGAACCGACCGATAAC<br>CCAAGCCCAAATACTCCATACT	cytochrome P450 reductase	GU808569.1	106	62
